# Identification of a *Neisseria gonorrhoeae* Histone Deacetylase: Epigenetic Impact on Host Gene Expression

**DOI:** 10.3390/pathogens9020132

**Published:** 2020-02-18

**Authors:** Susu M. Zughaier, Corinne E. Rouquette-Loughlin, William M. Shafer

**Affiliations:** 1 Department of Basic Medical Sciences, College of Medicine, QU Health, Qatar University, P.O. Box 2713, Doha, Qatar; 2 Laboratory of Bacterial Pathogenesis, Department of Veterans Affairs Medical Center, Decatur, GA 30033, USAwshafer@emory.edu (W.M.S.); 3 Department of Microbiology and Immunology, Emory University School of Medicine, Atlanta, GA 30322, USA; 4 The Emory Antibiotic Research Center, Emory University School of Medicine, Atlanta, GA 30322, USA

**Keywords:** *Neisseria gonorrhoeae*, HDAC, infection, epigenetic, H3K9ac, macrophage, survival, cytokines, chemokines, gonorrhea

## Abstract

Epigenetic reprogramming in macrophages is termed trained innate immunity, which regulates immune tolerance and limits tissue damage during infection. *Neisseria gonorrhoeae* is a strict human pathogen that causes the sexually transmitted infection termed gonorrhea. Here, we report that this pathogen harbors a gene that encodes a histone deacetylase-like enzyme (Gc-HDAC) that shares high 3D-homology to human HDAC1, HDAC2 and HDAC8. A Gc-HDAC null mutant was constructed to determine the biologic significance of this gene. The results showed that WT gonococci reduced the expression of host defense peptides LL-37, HBD-1 and SLPI in macrophages when compared to its Gc-HDAC-deficient isogenic strain. The enrichment of epigenetic marks in histone tails control gene expression and are known to change during bacterial infections. To investigate whether gonococci exert epigenetic modifications on host chromatin, the enrichment of acetylated lysine 9 in histone 3 (H3K9ac) was investigated using the TLR-focused ChIP array system. The data showed that infection with WT gonococci led to higher H3K9ac enrichment at the promoters of pro-inflammatory mediators’ genes, many TLRs, adaptor proteins and transcription factors, suggesting gene activation when compared to infection with the Gc-HDAC-deficient mutant. Taken together, the data suggest that gonococci can exert epigenetic modifications on host cells to modulate certain macrophage defense genes, leading to a maladaptive state of trained immunity.

## 1. Introduction

*Neisseria gonorrhoeae* is a strict human pathogen that causes the sexually transmitted infection termed gonorrhea. Importantly, gonorrhea is a major worldwide public health problem given its estimated yearly incidence of 87 million infections [1]. In addition to causing a high incidence of infection and disease, the gonococcus is noted for its capacity to develop resistance to antibiotics used in therapy [1]. In 2013, the Center for Disease Control declared antibiotic-resistant *N. gonorrhoeae* as an urgent threat to public health [2,3,4]. Recently, the World Health Organization placed *N. gonorrhoeae* on the high priority pathogen list for developing new antibiotics [5,6].

Gonococci can survive extracellularly and intracellularly, but, in both environments, the bacteria must adapt to pressures exerted by the host [7,8]. We reported that *N. gonorrhoeae* can survive in association with human monocytes and murine macrophages [9]. During infection of these phagocytes, it was noted that gonococci can enhance expression of iron-responsive genes encoding hepcidin (a master iron-regulating hormone), the antimicrobial protein termed NGAL and NRAMP1 while downregulating expression of the gene encoding the short chain 3-hydroxybutyrate dehydrogenase (BDH2) that catalyzes the production of the mammalian siderophore 2,5-DHBA involved in chelating and detoxifying iron. Based on these findings, we proposed that *N. gonorrhoeae* can subvert the iron-limiting innate immune defenses to facilitate iron acquisition and intracellular survival [7].

*N. gonorrhoeae* possesses several virulence factors that facilitate invasion and infection in human host. The addition of phosphoethanolamine (PEA) to lipid A by the enzyme PEA trasnferase, encoded by the phase-variable *lptA* gene [10], is important for bacterial resistance to cationic antimicrobial peptides [11] and complement-mediated killing by normal human serum [10,12]. PEA modification on lipid A enhanced bacterial survival within human polymorphonuclear leukocytes [13] and increased fitness of gonococci during experimental lower genital tract infection of female mice or in the urethra of human male volunteers [14,15]. Further, we recently reported that this PEA modification of lipid A reduced autophagy flux in macrophages, consequently delaying bacterial clearance and promoting intracellular survival [9]. Taken together, PEA-lipid A modification is a critical component in the ability of *N. gonorrhoeae* to evade host defenses and survive in macrophages.

The ability of gonococci to develop resistance to host AMPs prompted us to determine if this human pathogen might also modulate their production by phagocytes. In this respect, a previous report documented that live gonococci can downregulate cervical epithelial cell production of LL-37, a potent anti-gonococcal CAMP also produced by macrophages/monocytes and PMNs, to facilitate host cell invasion [16]. However, the mechanism by which gonococci downregulate host AMPs is unknown.

In order to explore the mechanism of *CAMP* gene suppression, we evaluated the potential impact of epigenetic factors. Although studies with other bacterial pathogens have documented the role of epigenetic factors, including histone deacetylases, it was heretofore unknown if gonococci can exert epigenetic modifications on host histones, thereby modulating host gene expression. Histones are highly basic proteins found in all eukaryotic cells and are required for packaging DNA in chromatin structures. Core histones have long tails that protrude from the nucleosome, which are targets for posttranslational modifications that consequently alter their interaction with DNA and nuclear proteins. Histone tail modifications include acetylation, methylation, phosphorylation, uniquitination, SUMOylation, citrullination and ADP-ribosylation [17]. These modifications influence various biological processes involved in DNA repair, gene regulation and cell division [17]. Several enzymes are involved in histone epigenetic modifications, including histone methyltranferases (HMT), histone acetyl transferases (HAT) and histone deacetylase (HDAC). The degree of lysine acetylation in core histone tails in particular directly influence transcriptional regulation, since acetylation reduces the positive charge on lysine, leading to reduced binding to the negatively charged DNA, thereby loosening chromatin structures facilitating transcription factors (TFs) binding to gene promoters. In contrast, deacetylation of lysine residues by HDACs increases the positive charges on histone tails that tighten its binding to DNA, rendering TFs binding sites inaccessible, resulting in gene suppression [18,19]. Against this background, we now report that gonococci (as well as commensal *Neisseria*) encode a highly conserved HDAC-like protein, herein named Gc-HDAC, that shares very high 3D homology to human HDAC1, HDAC2 and HDAC8. However, the function of this Gc-HDAC-like enzyme in gonococci is not known. We hypothesized that the Gc-HDAC-like protein exerts epigenetic modifications on host histones to suppress LL-37 and HBD-1 gene expression, which facilitates immune evasion and promotes intracellular survival. In this respect, we found that *N. gonorrhoeae* can exert epigenetic modifications on host chromatin where the epigenetic mark H3K9ac is highly enriched at the promoters of certain proinflammatory genes.

## 2. Results

### 2.1. Gonococcal Infection Downregulates Host Defense Peptides Expression in Macrophages

**Figure 1:**
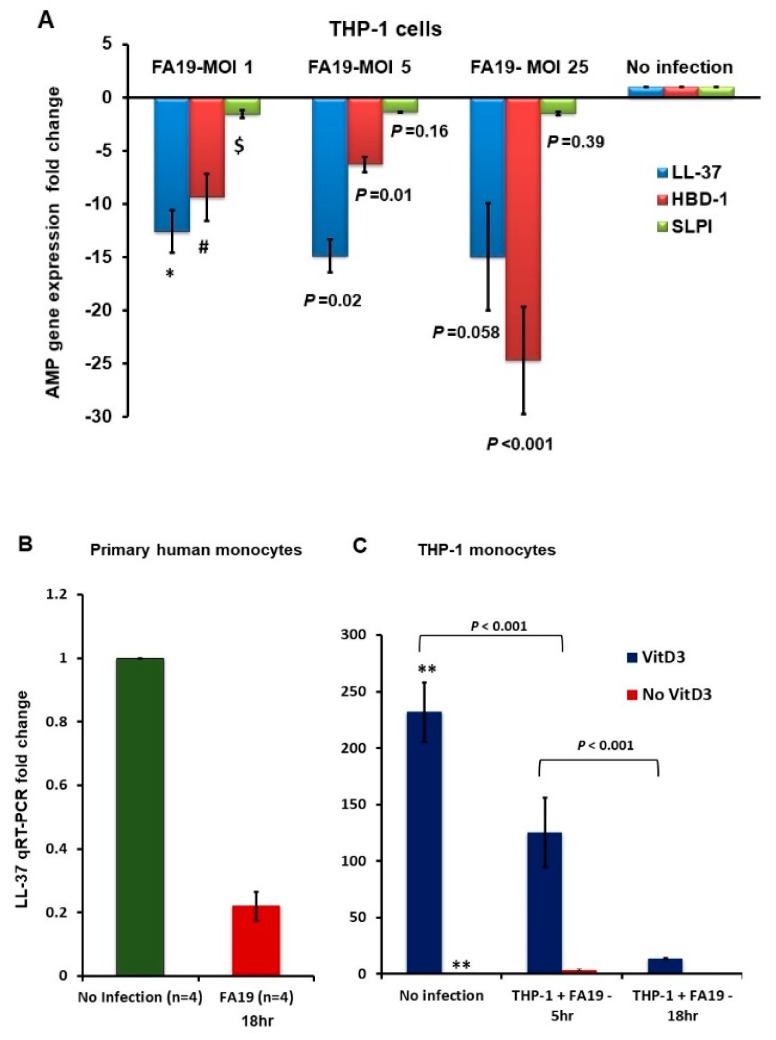
Gonococcal infection downregulates LL-37 and HBD-1 expression in monocytes. (**A**): Antimicrobial host defense peptide (AMP) gene expression in THP-1 cells infected with Gc strain FA19 overnight at multiplicity of infection (MOI) of 1, 5 and 25 measured by qRT-PCR. (**B**): LL-37 gene expression in primary human monocytes infected with Gc strain FA19 overnight at MOI of 10 measured by qRT-PCR. Peripheral monocytes were obtained from four different healthy donors. (**C**): Overexpression of LL-37 gene in human THP-1 monocytes treated with 10 nM of 1,25 dihydroxyvitamin D3 (VitD3) overnight prior to infection with Gc strain FA19 at MOI of 25. Downregulation of LL-37 gene expression was assessed at 5h and 18h post-infection. *p* values were calculated using a Student’s *t*-test in reference to noninfected cells (**). *p* values in reference to infection at MOI 1 for LL-37 expression (*), HBD1 (#) and SLPI ($). These data are representative of three independent experiments.

We previously reported that *Neisseria gonorrhoeae* survives in macrophages and induces robust cytokine and chemokine release [7]. In addition to its capacity to resist the action of antibacterial agents, including AMPs, we hypothesized that gonococci could influence expression of genes encoding host defensive responses. In support of this hypothesis, a previous report showed that gonococci can downregulate the expression of the human host defense AMP LL-37 in cervical epithelial cells for immune evasion [16]. Accordingly, in order to learn if gonococci could influence expression of host genes involved in innate immunity, we first investigated the expression of human AMPs LL-37, HBD1 and SLPI in THP-1 macrophage-like monocytic cells infected with live gonococcal strain FA19. The data demonstrated that gonococcal infection led to significant reduction in the expression of LL-37, HBD1 and SLPI compared to uninfected cells using quantitative RT-PCR (Figure 1A). We also investigated the expression of LL-37 in primary human peripheral monocytes obtained from healthy donors. We found that live gonococcal infection in primary human monocytes significantly reduced the expression of LL-37 when compared to uninfected cells (Figure 1B). Further, gonococcal infection also reduced LL-37 expression of human THP-1 cells, even when this gene was overexpressed by the addition of 10 nM of 1,25-dihydroxy vitamin D3, the active form of vitamin D3 (Figure 1C). Taken together, the data suggest that gonococcal infection of human macrophages can modulate host defense peptide expression. As will be described below, expression of host genes encoding cytokines and chemokines involved in innate host response to gonococcal infection can also be influenced by gonococci during infection.

### 2.2. Neisseria Gonorrhoeae Contains a Gene Encoding a Histone Deacetylase-Like (Gc-HDAC) Enzyme

**Figure 2:**
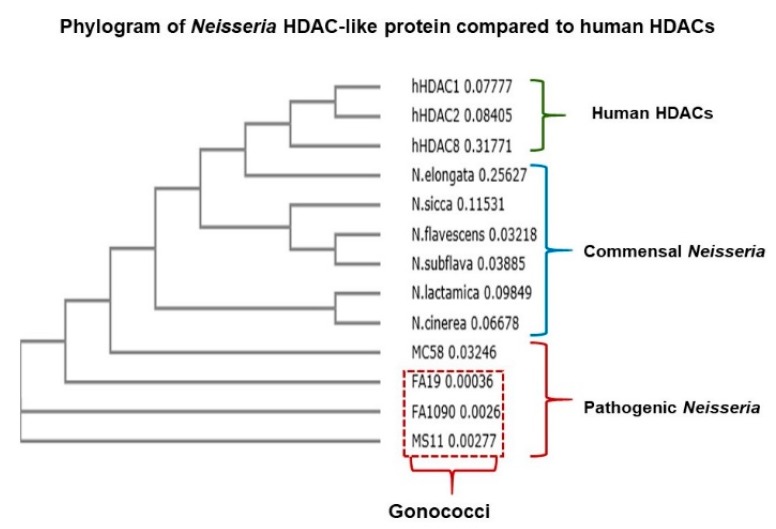
Evolution of *Neisseria gonorrhoeae* Gc-HDAC-like protein in human monocytes. Evolution of Neisseria Gc-HDAC-like protein compared to human HDACs. The multiple sequence alignment tool Clustal Omega was used to build the phylogenic tree (https://www.ebi.ac.uk/Tools/msa/clustalo/).

We hypothesized that the significant reduction in LL-37 gene expression in macrophages infected with live gonococci could be related to epigenetic modifications at the promoter of LL-37, resulting in decreased expression of the cognate gene. Accordingly, we performed a bioinformatics analysis of whole genome sequences from pathogenic and nonpathogenic *Neisseria spp*. searching for bacterial homologs of epigenetic modifying genes. Through this analysis, we detected an open reading frame (ORF) that could encode an HDAC-like enzyme. This ORF (termed *hdac*) was found in all pathogenic (*N. gonorrhoeae* and *N*. *meningitidis*) and commensal *Neisseria* species (*N. cinerea, N. lactamica, N. subflava, N. flavescens, N. sicca and N. elongata*); in gonococci, the ORF had been assigned NGO0187 in strain FA1090; NGEG_0305 in strain FA19 and NGK_0316 in strain NCCP11945 (https://blast.ncbi.nlm.nih.gov/Blast.cgi, https://www.kegg.jp/dbget-bin/www_bget?ngo:NGO0187 and https://www.genome.jp/dbget-bin/www_bget?ngk:NGK_0316, respectively). We termed the ORF as *hdac* as it is predicted to encode a highly conserved HDAC-like protein that shares 3D homology to human HDAC1, HDAC2 and HDAC8 (Figure 2). Currently, a total of 476 sequenced *Neisseria gonorrhoeae* strains have been found to contain this histone deacetylase protein homolog, which is reflected in the NCBI protein search (https://www.ncbi.nlm.nih.gov/protein).

### 2.3. Computational Analysis of Gc-HDAC Enzyme

**Figure 3:**
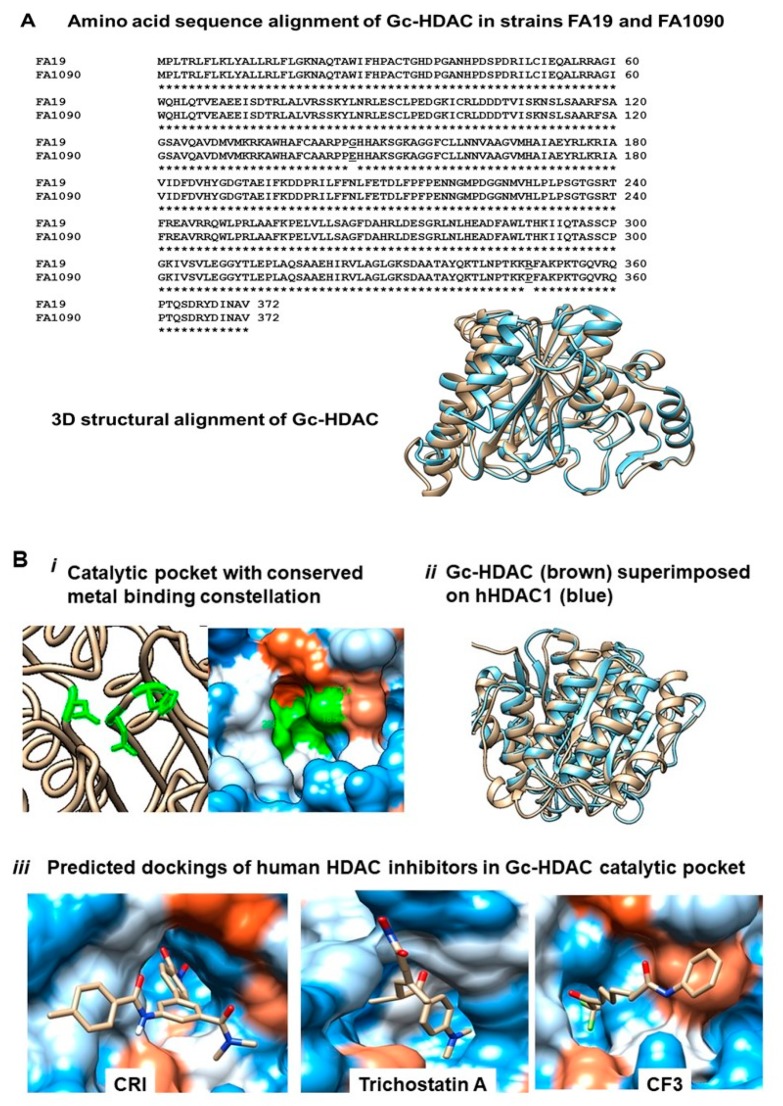
Computational analysis and in silico modeling of Gc-HDAC-like protein in *Neisseria gonorrhoeae*. (**A**): Amino acid sequence alignment of Gc-HDAC from gonococcal strains FA19 and FA1090 and their 3D structural alignment. (**B**): In silico modeling of Gc-HDAC-like protein: (i) predicted Gc-HDAC-like protein 3D structure revealing the catalytic pocket with the conserved metal binding constellation (green); (ii) predicted Gc-HDAC-like protein 3D structure (brown) is superimposed on human HDAC1 protein (blue) and (iii) predicted dockings of HDAC inhibitors CRI, trichostatin A and CF3 in Gc-HDAC catalytic pocket. Computational Gc-HDAC 3D protein structure and HDAC inhibitors dockings were predicted using I-TASSER, and PDB files were viewed using Chimera. The BS scores of top predictions for HDAC inhibitors CRI, TSA and CF3 are 1.51, 1.09 and 1.4, respectively. BS score definition by I-TASSER is a measure of local similarity (sequence and structure) between template binding site and predicted binding site in the query structure. Based on large-scale benchmarking analysis, a BS score >1 reflects a significant local match between the predicted and template binding site.

Computational analysis was performed on Gc-HDAC from gonococcal strains FA19, FA1090, MS11 and GD12. Computational modeling revealed that the protein has an active catalytic pocket containing the highly conserved zinc-binding triad (Asp185, His187 and Asp268) and shares high 3D homology to human HDAC1, HDAC2 and HDAC8 [20,21] and to bacterial HDLP from *Aquifex* and *Bordetella* [22]. Although the Gc-HDAC-like protein amino acid sequence homology to human and bacterial counterparts is relatively low (HDAC1 is 22%, HDAC2 is 19%, to human HDAC8 is 20% and to bacterial HDLPs is 29% and 30%, respectively), their 3D structural homology is remarkably high (Figure 3A). Furthermore, computational docking analysis using I-TASSER predicted that several HDAC inhibitors, such as trichostatin A (TSA); CF3 (9,9,9-trifluoro-8-oxo-*N*-phenylnonanamide); a fluorinated analog of SAHA; CRI (5-(4-methyl-benzoylamino)-biphenyl-3,4′-dicraboxylic acid 3-dimethylamide-4′-hydroxyamide) (Figure 3B) and B3N, also called M344 (4-(dimethylamino)-*N*-[7-(hydroxyamino)-7-oxoheptyl]benzamide), are able to bind to the catalytic core of the enzyme (Figure 3B). HDAC inhibitors compound structures are available at https://pubchem.ncbi.nlm.nih.gov/compound.

### 2.4. Expression of the Gonococcal HDAC-Encoding Gene

**Figure 4:**
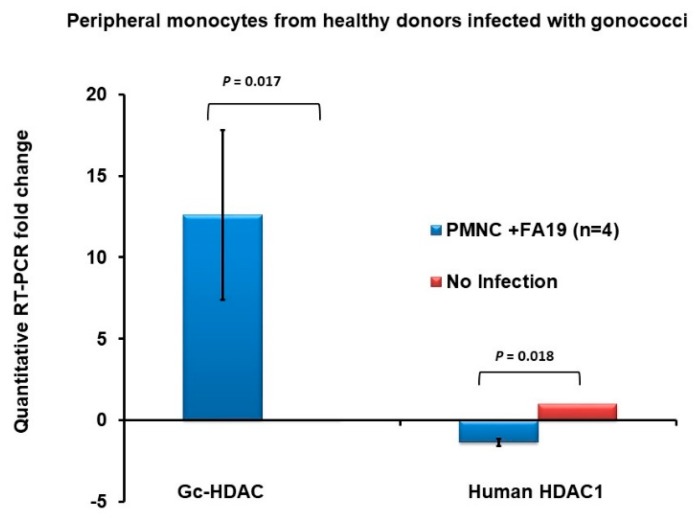
Expression of *Neisseria gonorrhoeae* Gc-HDAC-like protein during infection in peripheral human monocytes. Gc-HDAC gene expression during infection compared to human HDAC1 expression in peripheral human monocytes (PMNC) obtained from four healthy donors and infected with live gonococci at MOI 25 overnight. Gc-HDAC and hHDAC1 expression is assessed by quantitative RT-PCR normalized to β-actin gene expression and compared to noninfected PMNC (n = 4). *p* values were calculated using a Student’s *t*-test in reference to noninfected PMNC.

We examined whether the predicted *hdac* gene (GenBank: SCW18245.1; WP_050303785.1) could be expressed during growth in laboratory media and during infection of macrophages using qRT-PCR. The results showed that *hdac* is constitutively expressed at all growth phases (data not shown). To establish the biological significance of the predicted gene and whether it plays a role in pathogenesis, we examined its expression during infection of macrophages. Since Gc-HDAC has high 3D homology to human HDAC1 and peripheral monocytes express human HDAC1, we assessed the expression of both genes during infection of human peripheral monocytes. The results showed that the gonococcal *hdac* gene was expressed when monocytes from healthy donors were infected with live gonococcal strain FA19 at multiplicity of infection (MOI) of 10 (Figure 4). In contrast, gonococcal infection downregulated expression of the human HDAC1-encoding gene compared to uninfected monocytes (Figure 4). Therefore, we hypothesized that the Gc-HDAC-like protein may exert epigenetic modifications on host histones to suppress LL-37 gene expression or other determinants of innate immunity.

To determine whether the production of Gc-HDAC-like protein impacts its intracellular survival ability, we performed macrophage bactericidal assays. For this purpose, we employed WT strain FA19 and a genetic derivative containing an insertionally inactivated *hdac* (*hdac::spc*). We first examined if loss of the *hdac* gene altered the growth characteristics of *N. gonorrhoeae* strain FA19. The overall data from in vitro cultures indicate that the Gc-HDAC-deficient mutant has a slight growth defect (data not shown). We examined if Gc-HDAC protein affects survival in macrophages. The results showed that the *hdac*-null mutant was slightly attenuated in murine RAW264 macrophages compared to the WT parent strain (Figure S1A). Similar results were observed in human THP-1 cells (data not shown). Furthermore, bacterial Gc-HDAC was found to be expressed during infection in human THP-1 macrophage-like cells (Figure S1B). In contrast, human HDAC1 was downregulated in infected THP-1 cells (Figure S1B), but this was independent of the Gc-HDAC. The downregulation of HDAC1 in THP-1 was similar to the observed data in peripheral human monocytes (Figure 4). Although the data suggest that Gc-HDAC-like protein may facilitate intracellular survival, it is possible that the moderate growth defect of the mutant is responsible for its reduced intracellular survival in macrophages.

### 2.5. N. Gonorrhoeae Exerts Epigenetic Modifications on Host Innate Immune Genes in Infected Macrophages

**Figure 5:**
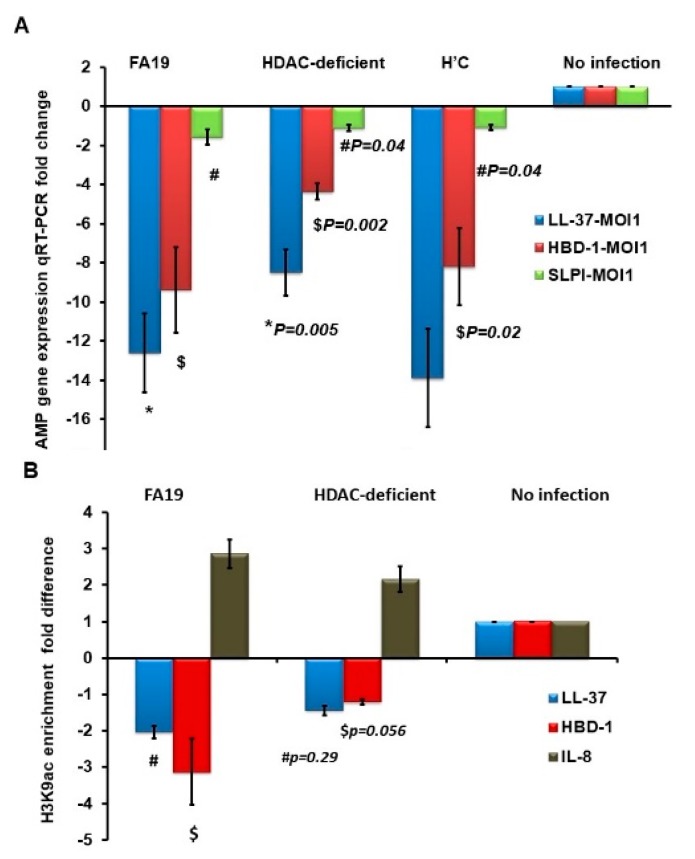
Expression of *Neisseria gonorrhoeae* AMPs gene expression in infected monocytes in the presence and absence of Gc-HDAC. (**A**): Expression of LL-37, HBD-1 and SLPI genes in THP-1 monocytes infected with WT strain FA19 or its isogenic Gc-HDAC-deficient mutant or the complemented H’C strain at MOI of 1 overnight (n = 3) was assessed using qRT-PCR. (**B**): H3K9ac epigenetic mark enrichment at the promoters of LL-37 and HBD-1 in THP-1 monocytes infected with WT strain FA19 or its isogenic Gc-HDAC-deficient mutant at MOI of 25 overnight (n = 3) was assessed using a ChIP assay. *p* values were calculated using a Student’s *t*-test in reference to cells infected with the WT FA19 strain for LL-37 expression (*), HBD1 (#) and SLPI ($).

**Figure 6:**
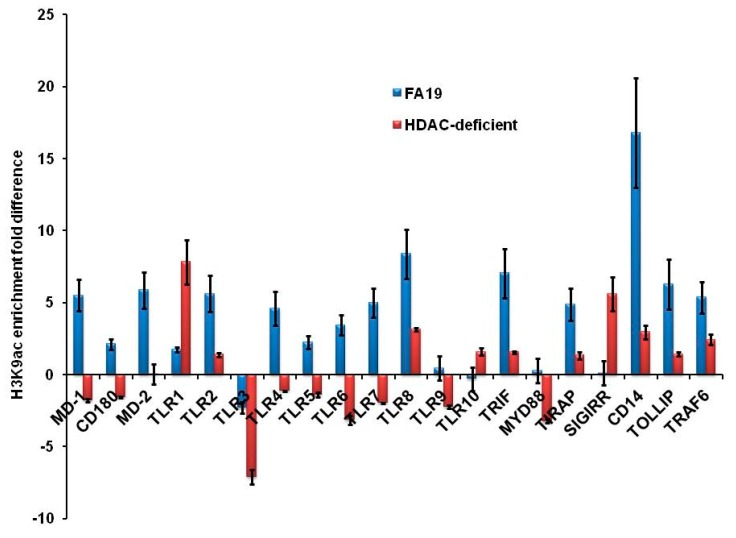
Gonococci exert epigenetic modifications in THP-1 monocytes. H3K9ac epigenetic mark enrichment at the promoters of genes involved in the TLRs signaling pathways. WT parent strain FA19: blue bars and isogenic HDAC-deficient mutant: red bars. Data are average of three independent ChIP experiments. *p* values were > 0.05 and were calculated using a Student’s *t*-test comparing WT FA19 to the HDAC-deficient mutant.

We hypothesized that the gonococcal HDAC-like protein could exert an epigenetic influence on host genes. Thus, potential acetylation of lysine could reduce histone binding to DNA and, therefore, allows transcription factors to bind to promoter elements, leading to gene regulation. In contrast, lysine deacetylation would lead to gene suppression. To examine whether gonococcal infection in macrophages causes epigenetic modifications, we performed a chromatin immune precipitation (ChIP) assay. Specifically, the alteration of histone 3 lysine 9 acetylation (H3K9ac) as a prominent epigenetic mark that changes during sepsis and infection in monocytes [23] was examined. We first examined the downregulation of human AMPs LL-37, HBD-1 and SLPI in THP-1 monocytes infected with live WT FA19, its isogenic Gc-HDAC-deficient and complemented strain. Using multiple MOIs (1, 5, 10, 25 and 50), we found that infection of target cells even at low MOI of 1 led to significant reduction in expression of AMP genes (Figure 5A). Importantly, reductions in gene expression show that AMPs were significantly downregulated in a Gc-HDAC-independent manner, although a slight but significant difference was observed when compared to parent strain FA19 (Figure 5A). This slight difference may be because the Gc-HDAC mutant has a moderate growth defect (see above). To examine the mechanism of CAMP gene downregulation, we then investigated epigenetic modifications at the promoters of host defense genes of LL-37 and HBD-1 during gonococcal infection with WT and the Gc-HDAC-deficient isogenic mutant. As expected, an H3K9ac epigenetic mark was not enriched at the promoters of host defense peptides LL-37 and HBD-1, suggesting gene silencing in a Gc-HDAC-independent manner (Figure 5B). Further, we investigated H3K9ac epigenetic mark enrichment in the promoters of other host innate immune genes involved in pathogen sensing and signaling; specifically, TLRs signaling pathways [24,25]. Results from the ChIP microarray showed that H3K9ac epigenetic mark is highly enriched in the promoters of proinflammatory and signaling genes of TLRs pathways in THP-1 cells infected with Gc-FA19 parent strain compared to its isogenic Gc-HDAC mutant or the noninfected THP-1 cells (Figure 6). Specifically, H3K9ac enrichment was observed in the promoters of the NFKB complex; other transcription factors like JNK, FOS and nuclear receptors; MAP kinases; TLRs and proinflammatory cytokines. H3K9ac epigenetic mark is highly enriched in the promoters of CD14, IL-10, type 1 IFN, TNFα and RELA 9p65 (Figure S2). Of note, the absence of Gc-HDAC differentially increased H3K9ac epigenetic mark enrichment in the promoters of IL-2, CCL2 (MCP-1), TLR1, SIGIRR, REL, MAPK8 and IKBKB. Most of these genes are negative regulators of the inflammatory response. Further, the observed epigenetic alterations at the promoters of host innate immune genes were confirmed by qRT-PCR using the TLR-focused RT2 microarray (Qiagen) to assess gene expression in infected macrophages (Figure S3). Taken together, the data suggest that Gc-HDAC-like protein in gonococci may contribute to histone modifications, consequently inducing proinflammatory genes while suppressing host defense peptides genes to facilitate survival and promote infection.

## 3. Discussion

Epigenetic reprogramming in macrophages is termed trained innate immunity, and it regulates immune tolerance and limits tissue damage during infection [26,27,28,29]. However, maladaptive states of trained immunity cause sepsis and hyper-inflammation. *N. gonorrhoeae* is known to evade multiple host defense systems to facilitate survival and promote infection. Here, we show that gonococcal infection of human macrophages led to significant reduction in host defense CAMP genes expression encoding LL-37, HBD-1 and SLPI. We also show for the first time that *N. gonorrhoeae* infection in macrophages can induce epigenetic modifications on host chromatin through a Gc-HDAC-independent process. Our data suggest that gonococcal infection in macrophages exerts epigenetic modifications to modify host gene expression. The maladaptive state of trained immunity impacts clearance of pathogens, as well as the development of optimal adaptive immune responses. Gonococcal infections are asymptomatic in 50%–70% of cases among females, as well as in some cases of male rectal gonorrhea [30,31]. However, gonococcal infections associate with severe pelvic inflammatory disease and infertility, in addition to increasing the risk of HIV transmission and other sexually transmitted infections like chlamydia [31]. Our data showed that gonococcal *hdac* is constitutively expressed at all growth phases. Interestingly, a recent study analyzed the complete gonococcal transcriptome response during anaerobic growth of gonococci and reported that an ORF identical to *hdac* was upregulated more than 4-fold compared to aerobic growth conditions [32]. This upregulation of gene expression under anaerobic conditions is physiologically relevant to gonococcal infections in fallopian tubes and upper genital tract causing pelvic inflammatory disease, which is a severe and symptomatic infection. Therefore, the Gc-HDAC-like protein may play a role in promoting ascending gonococcal infections. Further, gonococcal infections do not confer protective immunity following natural infections [33]. Therefore, we postulate that gonococcal infections induce a maladaptive state of trained immunity in the human host.

The exact molecular mechanisms by which gonococci exert epigenetic modifications are not clear. *N. gonorrhoeae* does not possess SET-containing domain effectors that are known to exert epigenetic modifications on host chromatin [34,35]. Using the bioinformatics approach, we identified a gene that encodes a histone deacetylase-like enzyme (Gc-HDAC) that shares high 3D homology to human HDAC1, HDAC2 and HDAC8. In eukaryotic cells, HDACs suppress gene expression by condensing chromatin packing consequently, preventing transcription factors from binding to gene promoters [21]. We tested our hypothesis, that the Gc-HDAC protein exerts epigenetic modifications on host histones to suppress LL-37 gene expression, which facilitates immune evasion and promotes intracellular survival. Of note, gonococcal infection in macrophages downregulated the expression of human HDAC1, the most commonly expressed human HDAC in myeloid cells [36,37,38,39], suggesting epigenetic modulation (Figure 4 and Figure S1B). This observation is novel and consistent with the notion that *N. gonorrhoeae* can modulate host responses for the purpose of immune evasion [16]. Although the Gc-HDAC mutant is attenuated, we concluded that the observed epigenetic modifications are Gc-HDAC-independent. Therefore, the exact molecular mechanism underlying these epigenetic modifications remains unclear. Bioinformatics analyses is ongoing to identify other potential effectors that *N. gonorrhoeae* may harbor that are directly causing these epigenetic modifications. Further, expanded epigenetic modifications profiling beyond epigenetic mark H3K9ac to investigate other epigenetic marks, such as H4K16 acetylation and H3K9 trimethylation [40], may shed a light on the possible role of Gc-HDAC in modifying host chromatin and promoting the gonococcal modulation of and evasion of host responses.

## 4. Materials and Methods

**Reagents:** RPMI 1640 medium, Dulbecco’s modified Eagle’s medium (D-MEM), fetal bovine serum (FBS), penicillin/streptomycin, sodium pyruvate and nonessential amino acids were obtained from Cellgro Mediatech (Herdon, VA). Chromatin immune precipitation ChIP reagents EpiTect^®^ ChIP kit and ChIP antibodies were purchased from Qiagen (Hilden, Germany). HDAC inhibitors trichostatin A and sodium butyrate TSA were purchased from Sigma (St Louis, MO, USA). Other HDAC inhibitors valproic acid and entinostat were kind gifts from Dr. Seth Brodie (Winship Cancer Center, Emory University School of Medicine, Atlanta, GA, USA).

**Computational analysis:** A bioinformatics blast search for conserved SET or HDAC domains was performed on all available *Neisseria spp.-*sequenced genomes using the “Delta Blast” search function available by the National Center for Biotechnology Information, known as NCBI (https://blast.ncbi.nlm.nih.gov/Blast.cgi?PROGRAM=blastp&PAGE_TYPE=BlastSearch&BLAST_PROGRAMS=deltaBlast&RESET_PROGRAM=on&RID=W4K9E36F014). We employed computational modeling to predict the GC-HDAC-like protein structure-function. The amino acid sequence of the identified GC-HDAC-like protein sequence of 372 amino acids was deposited into two different molecular modeling softwares, I-TASSER [41] and Phyre Protein Fold [42], to predict protein 3D structure. The predicted protein databank (PDB) files were then visualized using Chimera software [43]. Computational analysis was performed on GC-HDAC from gonococcal strains FA19, FA1090, MS11 and GD12.

The following HDAC inhibitors were predicted to dock in the Gc-HDAC enzyme catalytic pocket: CF3: 9,9,9-TRIFLUORO-8-OXO-N-PHENYLNONANAMIDE; CRI: 5-(4-METHYL-BENZOYLAMINO)-BIPHENYL-3,4′-DICARBOXYLIC ACID 3-DIMETHYLAMIDE-4′-HYDROXYAMIDE and TSA: trichostatin A. HDAC inhibitors compound structures are available at https://pubchem.ncbi.nlm.nih.gov/compound.

**Construction of genetic derivatives:** The *hdac* gene was amplified using primers 0187pac1 (5′-GATCTTAATTAATATGCCGTCTGCACCCCC-3′) and 0187pme1 (5′- GATCGTTTAAACGAAAACCGAATCGGCTTCAG -3′) and FA19 genomic DNA as the template. The corresponding 1118 bp PCR product and the pGCC4 vector [44] were digested by Pac1 and Pme1 and then ligated overnight at 16 °C. The ligation was then transformed into *Escherichia coli* DH5α. Transformants were verified by PCR, and a verified transformant was selected for further study. After growth overnight, DNA from the transformant was extracted using a Qiaprep column, as described by the manufacturer (Qiagen), and its insert was sequenced. pGCC4 *hdac* was digested by Xho1, and a spectinomycin (Spc) cassette was inserted in the Xho1 site. The ligation was transformed into *E. coli* DH5α, and transformants were selected on GC plates supplemented with 60 µg/mL of Spc and verified by PCR. After growth overnight, pGCC4*hdac::spc* was extracted using a Qiaprep column, as described by the manufacturer (Qiagen). A PCR was performed using primers 0187pac1 and 0187pme1 and pGCC4*hdac::spc* as the template. This 3118 bp PCR product was then transformed into FA19, as previously described [45]; transformants were selected on GC plates supplemented with 60 µg/mL of Spc. FA19*hdac::spc* transformants were verified by PCR. One clone was selected and transformed by pGCC4*hdac*; transformants were selected on GC plates supplemented with 1 µg/mL of erythromycin (ery). One FA19*hdac::Spc*C′ clone was selected, and the insertion of the wild-type copy of *hdac* at the *lctP/aspC* locus was verified by PCR and sequencing.

**Construction of FA19Str^R^*hdac::Spec* and FA19Str^R^*hdac::Spc*C’:** DNA from strain FA19Str^R^ [46] was extracted [47] and transformed into FA19*hdac::spc* and FA19*hdac::Spc*C′, as previously described [45]. Transformants were selected on GC plates supplemented with 1600 µg/mL of streptomycin. Four transformants from each transformation were selected, and the *rpsL* gene was PCR-amplified using primers rpsLF (5′-CGTTATGCTTGACTGTCTGC-3′) and rpsLR (5′-TCTATTCCCATGAATACCCAAT-3′) and sequenced.

**Bacterial growth curves:** To investigate whether the deletion of HDAC gene impacts bacterial growth, the growth rate of the parent strain FA19 to the HDAC-deficient isogenic and complemented mutants were compared, as previously described [24]. Briefly, WT strain FA19 (wild-type; WT), its isogenic mutant *hdac::spc* strain and complemented strain (HDAC-C’) strains were grown as pilus-positive, opacity-negative colony variants on GC agar containing defined Supplements I and II and 1 mM isopropyl β-D-1-thiogalactopyranoside (IPTG) under 5.0% (*v*/*v*) CO_2_ at 37 °C, as described by Shafer et al. [45]. Gonococci were grown in GC broth with supplements and 0.043% (*w*/*v*) sodium bicarbonate at 37 °C in a shaking water bath. The viability of Gc cultures was determined using dilution plating onto GC agar, and colony forming units (CFU) were enumerated after 24 h of incubation at 37 °C in a CO_2_ incubator. Gc grown on agar plates were resuspended in GC broth and harvested by centrifugation at 5000× *g* for 10 min. The bacterial pellet was washed twice with PBS and resuspended in 10 mL of D-MEM tissue culture medium without antibiotics to prepare a live Gc inoculum for macrophage infection experiments (see below) [7].

**Cell cultures**: THP-1 human macrophage-like monocytic cells were obtained from the American Type Culture Collection (ATCC, Manassas, VA) and grown in RPMI 1640 with L-glutamate supplemented with 10% (*v*/*v*) FBS, 50 IU/mL of penicillin and 50 µg/mL of streptomycin. Culture flasks were incubated at 37 °C with humidity and 5% (*v*/v) CO_2_. Murine macrophages (RAW264 from ATCC) were grown in D-MEM supplemented and incubated as noted above.

**Macrophage infection assay:** Freshly grown human THP-1 macrophage-like monocytic cells (in the absence of antibiotics) were adjusted to one million cells/mL, then transferred into 8-well tissue culture plates (2 mL/well) and infected with live Gc FA19, Gc-*hdac*::*spc* or its complemented strain at multiplicity of infection (MOI) of 25, 10, 5 and 1, then incubated overnight at 37 °C with 5% (*v*/*v*) CO_2_. Uninfected cells in triplicate wells were also incubated simultaneously and were used as a minus infection control. Supernatants from infected or uninfected macrophages were harvested and saved at −20 °C for determination of chemokines release, and cells were washed with PBS, pelleted (1000× *g* for 5 min) and saved at −80 °C for Western blot analysis.

**Isolation of peripheral monocytes:** We previously published the detailed protocol of isolating peripheral monocytes from healthy donors in our previous paper [7]. The study was deemed exempt from the Institutional Review Board (IRB) at Emory University since peripheral monocytes were completely de-identified without any link to donors’ identification. Briefly, whole blood (15 mL with EDTA) was collected from healthy donors after obtaining written informed consent under Emory University IRB approval to collect healthy donors’ plasma for other unrelated studies. Peripheral monocytes were isolated using Ficoll-density gradient centrifugation (Histopaque 1077, Sigma-Aldrich, St Louis, MO, USA), as described [7]. Primary peripheral monocytes were infected with live GC-FA19 at an MOI of 10 and incubated overnight. Monocytes were harvested for RNA isolation, and gene expression was measured by quantitative RT-PCR.

**Macrophage bactericidal assay:** To determine whether the Gc-HDAC protein plays a role in the survival of Gc in association with macrophages, we employed murine RAW264 macrophage bactericidal assays, as previously described [7,9]. Briefly, freshly grown Gc strains were adjusted to an OD600 of 1.0 (~1 × 10^8^ CFU/mL) in antibiotic-free D-MEM medium containing 10% heat-inactivated fetal bovine serum (FBS). Macrophages were also freshly grown, washed and adjusted to 1 million cells/mL in antibiotic-free D-MEM medium containing 10% FBS. Since these RAW264 macrophages are adherent, cells were seeded in 24-well tissue culture plates (1 million cells/well) and allowed to adhere overnight prior to infection with live gonococci at an MOI of 25, as described above. After one hour of initiated phagocytosis at 37 °C, adherent RAW264 cells were washed three times with antibiotic-free medium containing 10% heat-inactivated FBS, and all fluids were carefully removed without disturbing the adherent macrophages. One ml of fresh antibiotic-free medium containing 10% heat-inactivated FBS was added to each well, and infected cells were further incubated for 1 h. Extracellular Gc were removed by washing adherent macrophages three times with D-MEM medium. Viable intracellular (or tightly adherent) Gc were assessed by serial plating of macrophage cultures lysed using 0.01% triton X-100 in PBS, as previously described [7].

**ChIP assay:** To investigate whether *N. gonorrhoeae* exerts epigenetic modifications on host histones, a chromatin immune precipitation (ChIP) assay was performed. Briefly, histone modification patterns in the 1000 bp proximal promoter regions of LL-37 and HBD-1 genes were analyzed by ChIP and qPCR [48]. THP-1 cells were infected with gonococci at an MOI of 25, as described above. All cells were incubated for 16 h and cross-linked with 1% formaldehyde. THP-1 cells were lysed with lysing buffer (50 mM Tris–HCl, pH 8.0, 10 mM EDTA, 1% (wt/vol) SDS, protease inhibitor cocktail (1:100 dilution from 100× stock, Thermo Scientific, Hanover Park, IL, USA), 1 mM 189 PMSF, 20 mM Na-butyrate), and nuclei was harvested by centrifugation at 4000× *g* for 5 min at 4 °C. Chromatin was sheared on ice with a Microson Ultrasonic Cell Disruptor, XL (Heat System, Newtown, CT, USA). Specific histone modifications antibodies: anti-acetyl-Histone H3 (Lys9) (Cat# 07-352), anti-trimethyl-Histone H3 (Lys9) (Cat# 07-442) or anti-trimethyl-Histone H3 (Lys4) (Cat# 07-473) (all from Millipore, Hayward, CA, USA) conjugated to protein A /G Dynabeads (Life Technologies, Carlsbad, CA, USA) were used to pull down modified histone bound to DNA. These specific antibodies were incubated with sheared chromatin at 4 °C for 2 h. Isotype-matched antibody was included as negative ChIP control. The precipitated histone DNA was subjected to proteinase K digestion (50 µg/mL) for 30 min at 56 °C. DNA was purified using Qiaquick nucleotide removal columns from Qiagen (Hilden, Germany). The harvested DNA was quantified by qPCR using primers specific for the LL-37 (*camp*) and HBD-1 (*hbd-1*) gene promoter regions. The following primers were used in ChIP PCR quantitation: c-LL-37-F: 5’-GGCTTGGGAACATTTTGAGA-3’ and c-LL-37-R: 5’-ATCCCCTTCTGCATCCTTCT-3’ and c-HBD-1-F: 5’-TCCAGAAACCCCATCAGAAC-3’ and c-HBD-1-R: 5’-CCGCTGGATTTAGCTTTCAG-3’. The relative fold change of DNA was calculated by comparing the percentage of precipitated DNA (% input) in infected THP-1 cells to that in the uninfected control cells. Antibodies used in the ChIP assay are specific to histone modifications, such as acetylated and trimethylated lysine residues in histone tails.

**Host Cell gene expression using TLR-focused microarray (RT^2^ Real-Time PCR array):** One million cell/mL THP-1 cells were transferred to 12-well formats and then infected with *N. gonorrhoeae* strain FA19 or its Gc-HDAC isogenic mutant at MOI of 1, 10 and 15. Uninfected cells were used as controls for basal gene expression level. Cells were further incubated overnight at 37 °C under 5% CO_2_. RNA was isolated using RNeasy Mini kits (Qiagen, Hilden, Germany), as previously described [49]. Briefly, cells were harvested in RLT buffer, passed over QiaShredder columns, and the resulting lysate was mixed in 70% ethanol, then was passed over RNeasy columns (Qiagen). Isolated RNA was then reverse-transcribed to cDNA using a First Strand kit from Qiagen. The generated cDNA was diluted with 91 µL of ddH_2_O to each 20 µL of cDNA synthesis reaction. The experimental cocktail for real-time PCR was prepared in a sterile boat as follows: 1275 µL of 2X SYBR Green PCR Master Mix (Applied Biosystems, Foster City, CA, USA), 102 µL of diluted cDNA and 1173 µL of ddH_2_O. Real-time PCR was then performed using RT^2^ Profiler^TM^ PCR Array (Qiagen) in 96-well format pre-loaded with the primers. Human Toll-like receptor signaling pathway and human apoptosis pathway RT^2^ Profiler^TM^ PCR Arrays profile the expression of 84 genes related to the TLR-mediated signal transduction pathway. In addition to primers, the array contains all positive and negative controls required for the real-time PCR procedure. To start the real-time PCR reaction, 25 µL of experimental cocktail mix was carefully added to each well in the RT^2^ PCR Array using a multichannel pipette, then was tightly sealed with the optical adhesive film. The PCR parameters were set as follows: 2 min at 50 °C, 10 min at 95 °C and 45 cycles of 95 °C for 15 s, followed by 1 min at 62 °C. For data analysis, the Excel-based PCR Array data analysis template (downloaded from this link: https://www.qiagen.com was used. Gene expression profiles were automatically calculated from threshold cycle data generated from the real-time instrument, and any C_t_ value equal or greater than 35 was considered negative.

**Quantitative RT-PCR analysis:** RNA was extracted from infected and uninfected macrophages using an RNeasy kit and was transcribed to cDNA using a reverse transcriptase kit from Qiagen, as previously described [7]. The relative gene expression was normalized to uninfected controls, and primers used in qRT-PCR were previously described [7]. For this study the following primers used in qRT-PCR were: LL-37-F: GGGCACACTGTCTCCTTCAC and LL-37-R: TCGGATGCTA**A**CCTCTACCG. The following primer sets (Quantitect^®^ Primer Assay) were purchased from Qiagen: human HBD-1 (Hs_DEFB_1_SG), human SLPI (Hs_SLPI_1_SG), human HBD-5 (Hs_DEFB105A_3_SG) and hHDAC1 (Hs_HDAC1_1_SG).

**Cytokine and Chemokine release quantification:** In order to determine whether the production of Gc-HDAC impacted the magnitude of the innate immune response, cytokines and chemokines released from infected macrophages were quantified. Briefly, freshly grown human THP-1 monocytic cells were harvested and adjusted to one million cells/mL without antibiotics, transferred into 6-well tissue culture plates (3 mL/well) and infected with viable strains FA19, Gc-HDAC::spc and the complemented strain Gc-HDAC’C at a multiplicity of infection (MOI) of 1, 5, 10 and 25. Cells were then incubated at 37 °C with 5% CO_2_ for 5 h or overnight. Uninfected cells in triplicate wells were also incubated simultaneously and used as a no-infection control. Supernatants were harvested and saved at −20 °C for determination of cytokine release, and cells were collected for RNA extraction. Cytokine/chemokine releases in the supernatants collected from infected macrophages were assessed using the multiplex ELISA panel from Invivogen (San Diego, CA, USA), and the following cytokines: TNFα, IL-1β, IL-6, IL-4, IL-12p70, IL-17, IL-10, IFNγ and IFNα and chemokines: IL-8, IP-10, MCP-1, RANTES, Eotaxin, MIP1α and MIP1β were included. Briefly, 50 µL of supernatants were mixed with the multiplex magnetic beads precoated with cytokines and chemokines-specific antibodies in buffer, and ELISA was performed using the Luminex array.

**In vitro fitness analysis of gonococci:** Broth cultures (25 mL GC broth (GCB) supplemented with Kellogg’s supplements I and II and 5 mM NaHCO_3_) of wild-type [29] parent FA19 or Gc-HDAC-deficient mutant strains (passaged once) were prepared at an OD_600_ of 0.05. A third broth culture with an equal mixture of both parent and mutant strains (i.e., competitive culture) was also prepared with the same starting OD_600_. Over an 8-h period, optical density of the cultures was measured every hour, while bacterial counts were assessed every 2 h using plate dilution. For the latter, plates were incubated at 37 °C with 7% CO_2_ for 48 h before counting CFUs. The competitive index was calculated using the following equation: output ratio divided by input ratio, where the output ratio is the number of mutant CFU divided by those from the WT parent CFU at a particular time point, while the input ratio is the number of mutant CFU divided by the WT parent CFU in the inoculum (T = 0) of the competitive culture.

**Statistical analysis**: Mean values ± SD (standard deviation) and *p* values (Student’s *t-*test) of at least three independent determinations were calculated with Microsoft Excel software.

## Supplementary Materials

The following are available online at https://www.mdpi.com/2076-0817/9/2/132/s1: Figure S1: Survival of gonococcal strain FA19 and its isogenic Gc-HDAC deficient mutant in macrophages. Figure S2: Gonococci exert epigenetic modifications in THP-1 monocytes. Figure S3: Pro-inflammatory genes expression is highly upregulated in macrophages infected with live gonococci.
