# Supplementary material for: Identification of a Neisseria gonorrhoeae Histone Deacetylase: Epigenetic Impact on Host Gene Expression
Source: Pathogens. 2020 Feb 18;9(2):132. doi: 10.3390/pathogens9020132 (PMC7168274; doi:10.3390/pathogens9020132)
Supplement: Supplementary file 1 [file pathogens-09-00132-s001.pdf]

pathogens

MDPI

Article

Identification of a *Neisseria gonorrhoeae* Histone Deacetylase: Epigenetic Impact on Host Gene Expression

Susu M. Zughaier [1,*], Corinne E. Rouquette-Loughlin [2,3] and William M. Shafer [2,3,4]

[1] Department of Basic Medical Sciences, College of Medicine, QU Health, Qatar University, PO Box 2713, Doha, Qatar

[2] Laboratory of Bacterial Pathogenesis, Department of Veterans Affairs Medical Center, Decatur, GA 30033, USA; corinne@patweb.com (C.E.R.-L.), wshafer@emory.edu (W.M.S.)

[3] Department of Microbiology and Immunology, Emory University School of Medicine, Atlanta, Georgia 30322, USA

[4] Antibiotic Research Center, Emory University School of Medicine, Atlanta, Georgia 30322, USA

* Correspondence: szughaier@qu.edu.qa; Tel.: +974 4403 7859

Received: 14 January 2020; Accepted: 14 February 2020; Published: date

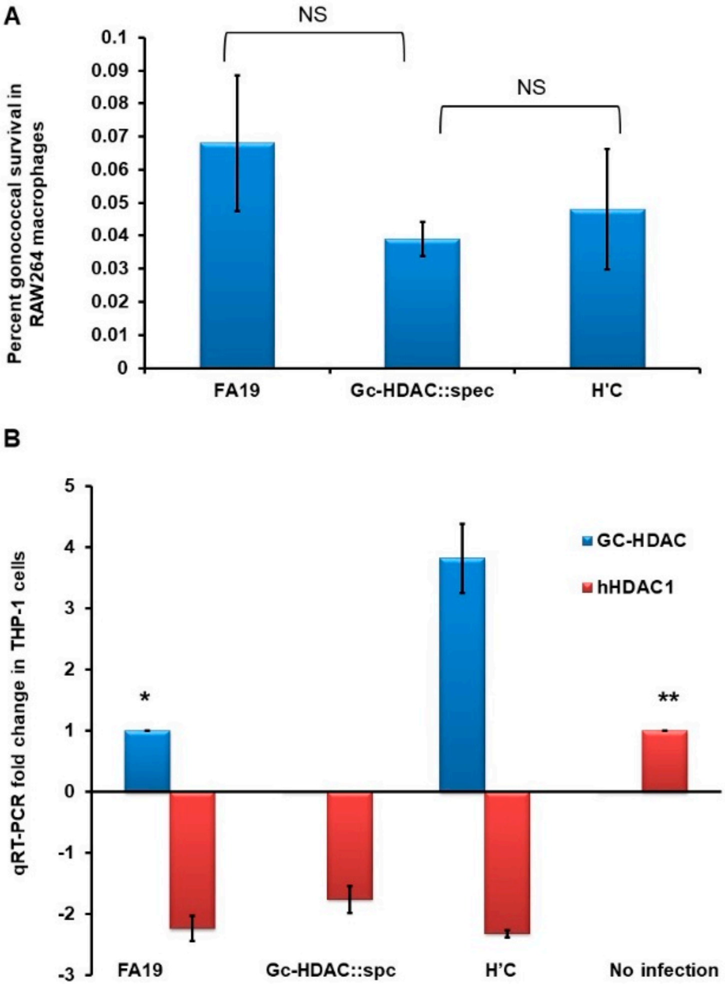

Figure S1. Survival of gonococcus strain FA19 and its isogenic Gc-HDAC-deficient mutant in macrophages. **A**: Intracellular survival of the Gc-HDAC-deficient (Gc-HDAC::spc) mutant in murine

macrophages compared to parent strain FA19 and the complemented mutant H'C. RAW264 macrophages were infected with live gonococci at an MOI of 25, and bacterial survival was assessed 2 h post phagocytosis (n=4). *p* values were calculated using a Student's *t*-test in reference to parent strain FA19; NS: not significant. **B**: Upregulation of bacterial Gc-HDAC gene expression in gonococci associated with THP-1 cells during infection. THP-1 cells were infected with live gonococcal strains at an MOI of 25 for 16 h. RNA was isolated, and bacterial *hdac* expression (normalized to 16S rRNA) was assessed using qRT-PCR. As a control, human *hdac1* gene expression normalized to β-actin was also assessed using qRT-PCR. Error bars represent ±SD from the mean of triplicate readouts from at least 3 independent experiments. *p* values were > 0.01 and were calculated using a Student's *t*-test for Gc-HDAC (*) and hHDAC1(**) expression in reference to no infection.

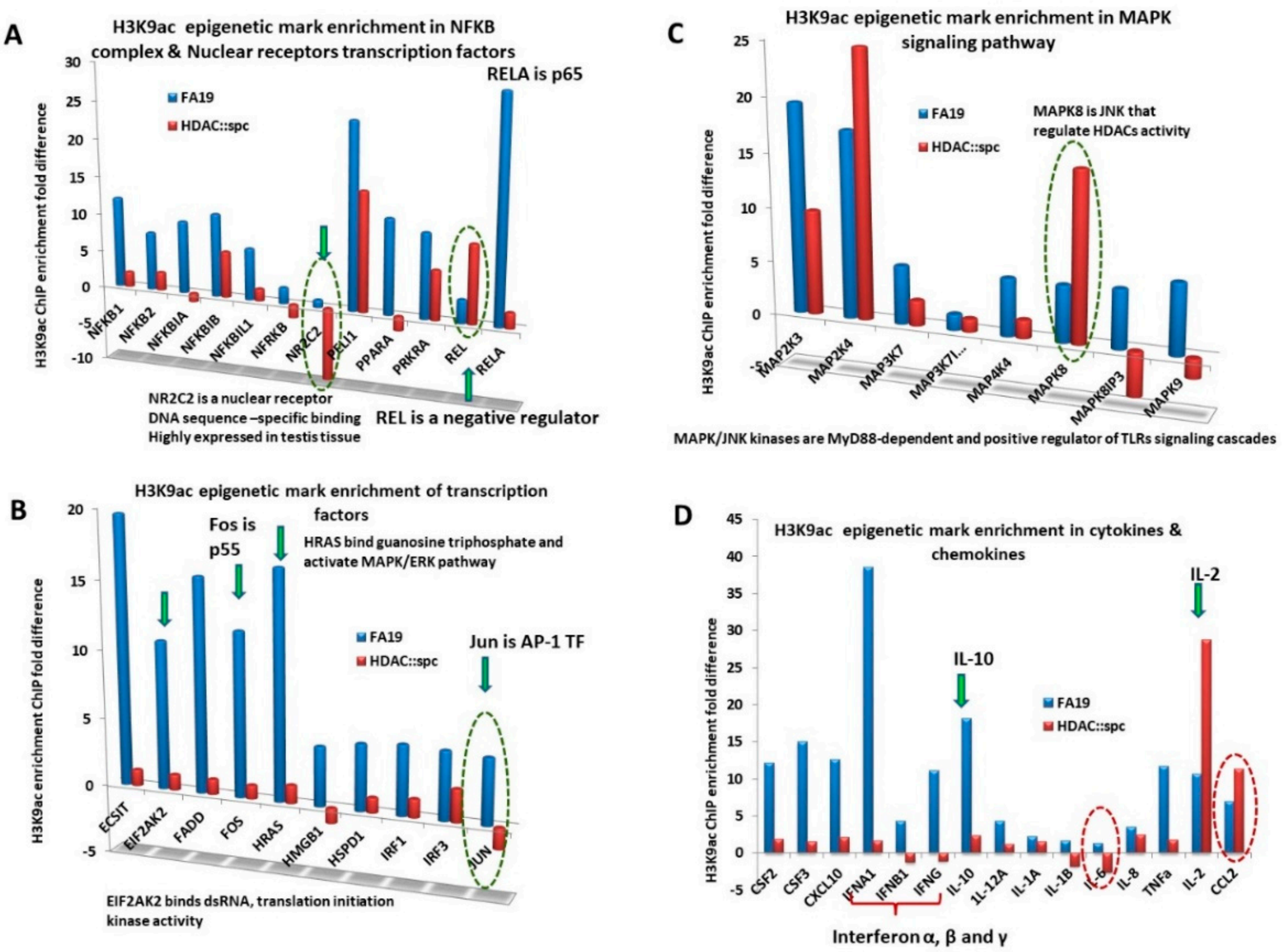

Figure S2. Gonococci exert epigenetic modifications in THP-1 monocytes. ChIP TLR-focused arrays were used to investigate H3K9ac epigenetic mark enrichment at the promoters of genes involved in the TLRs signaling pathways. Human monocytic THP-1 cells infected with Gc-FA19 at an MOI of 25 overnight were compared to uninfected cells detected using a TLR pathway-focused ChIP qPCR array in a 96-well plate. Panels represent: **A**: H3K9ac enrichment in promoters of NFkB and nuclear transcription factors. **B**: H3K9ac enrichment in promoters of various transcription factors. **C**: H3K9ac enrichment in promoters of the MAPK signaling pathway. **D**: H3K9ac enrichment in promoters of cytokines and chemokines. WT parent strain FA19: blue bars and isogenic HDAC-deficient mutant: red bars.

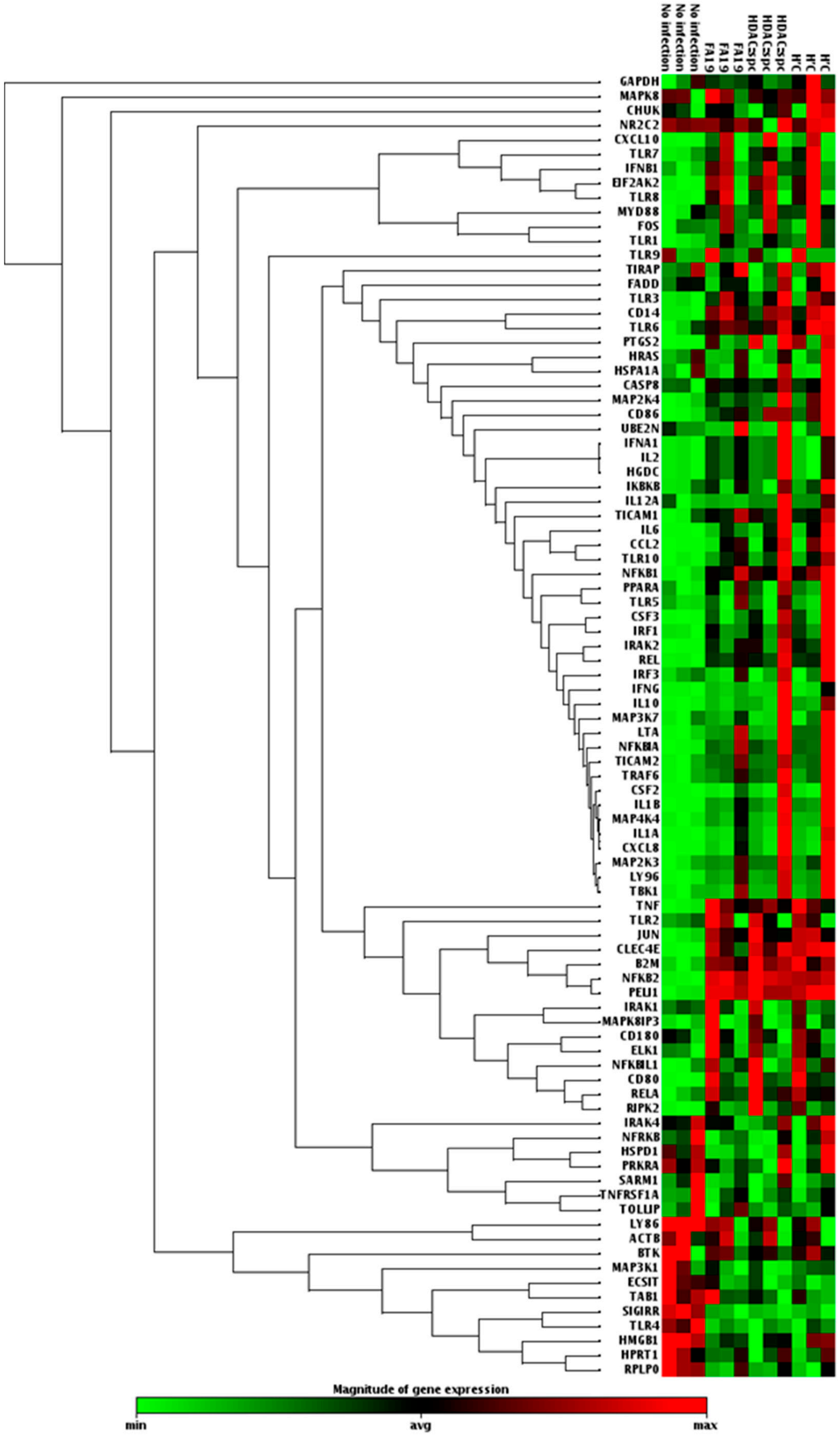

Figure S3. Pro-inflammatory genes expression is highly upregulated in macrophages infected with live gonococci. Host gene expression in THP-1 macrophages infected with gonococci parent strain

FA19 and its isogeneic HDAC-deficient mutant and the complemented mutant (H'C) at MOI 25. Real-time PCR was then performed using RT2 Profiler™ PCR Array (Qiagen) in 96-well format pre-loaded with the primers. Human Toll-like receptor signaling pathway and human apoptosis pathway RT2 Profiler™ PCR Arrays profile the expression of 84 genes related to TLR-mediated signal transduction pathway. Data are presented in a heat map.

© 2020 by the authors. Submitted for possible open access publication under the terms and conditions of the Creative Commons Attribution (CC BY) license (http://creativecommons.org/licenses/by/4.0/).
